# Techno-functional, physicochemical and thermal characteristics of black chickpeas aquafaba under ultrasound pre-processing

**DOI:** 10.1016/j.heliyon.2024.e40149

**Published:** 2024-11-09

**Authors:** Fatemeh Roosta, Abdollah Hematian Sourki

**Affiliations:** Department of Food Science and Technology, Faculty of Agriculture, Jahrom University, PO Box: 74135-111, Jahrom, Iran

**Keywords:** Aquafaba, Black chickpeas, Liquid gold, Foaming, Soluble protein, Thermogravimetric

## Abstract

Aquafaba is the liquid that remains from the cooking of beans in the canning industry, generally discarded as wastewater. This research aimed to optimize ultrasound pretreatment to enhance this by-product and introduce it as a high-added value product (known as liquid gold) in the food industry. The results showed that with the increase in the sonication time and amplitude, the extraction efficiency, soluble protein content, the density, and dry matter content of black chickpeas aquafaba increased significantly (p < 0.05). The results also demonstrated that foaming ability (in short ultrasonication times) and foam stability significantly increased with higher amplitude. The results of numerical optimization showed that ultrasound pre-treatment for 30 min with an amplitude of 72 % on black chickpeas before the cooking process created the best conditions for aquafaba extraction. Under these optimized conditions, the results yielded the highest values so that extraction efficiency, protein content, density, dry matter, foaming ability, and foam stability were 212.07 %, 3546.7 mg/kg, 1.038 g/mL, 4.76 %, 243.57 %, and 44.50 % respectively. Thermogravimetric and FT-IR analysis showed that the pre-sonication process not only makes aquafaba thermally stable, but also does not cause any structural changes in its chemical components. According to the favorable physicochemical characteristics of aquafaba, this product can benefit the food industry. It can be a material with high added value, profitable for the canning industry.

## Highlights

• Ultrasound-assisted extraction significantly increased the extraction efficiency and protein content of aquafaba.
• Ultrasonic pre-processing improved the techno-functional properties of black chickpeas aquafaba.
• The pre-sonicated aquafaba did not contain harmful or unknown compounds.
• Ultrasonic pre-processing improved the thermal stability of black chickpeas aquafaba.

## Graphical abstract

**Figure:**
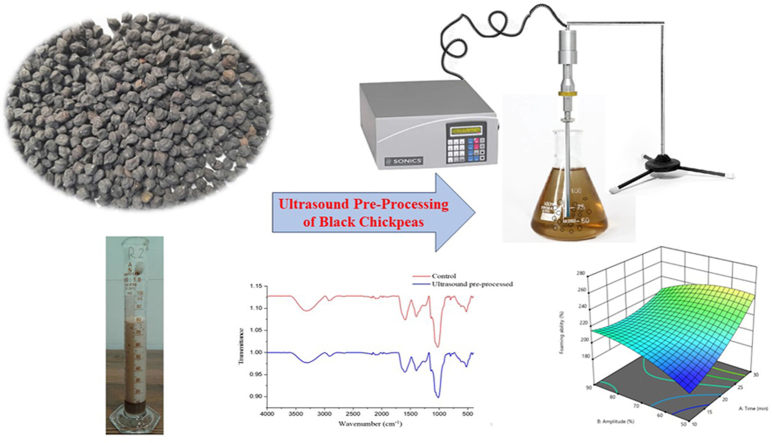

## 1 Introduction

During the processing and cooking of legumes, a semi-viscous liquid called "aquafaba" emerges from the cooked legumes and is commonly known as a waste product in food processing [1,2]. Aquafaba, or the extract obtained from the cooking of legumes, has many functional properties. It can assist in foam production, emulsification, binding, and thickening, which makes it useful in many food recipes, e.g., meringue recipes, mayonnaise, vegan cakes, butter, whipped cream, etc. [3]. Aquafaba's functional properties, including fat binding, water retention, solubility, and its ability to form gels, foams, and emulsions, make legume proteins a viable alternative to animal proteins in various food products like salad dressings and chicken nuggets [4,5]. In addition to these nutritional and functional benefits, it has low allergic properties that enable stable production, low price, and high production volume. Thus, the industrial use of proteins from legumes and pulses is preferable [6]. The added value of aquafaba is so high that it has earned the name "liquid gold" in the food industry.

Black chickpeas (Cicer arietinum L.) are an annual plant from the legume family. It grows in a wide range of climatic conditions and subtropical regions. In addition to the importance of this plant as a food source for human and animal nutrition, it helps in soil fertility management [7]. Farmers traditionally plant black chickpeas in Apulia (southern Italy) and northwestern regions of Iran [8]. Costantini et al. [9] reported that black chickpeas have a higher nutritional value than common chickpeas. Black chickpeas have higher carbohydrates, protein, fat, soluble sugars, total phenolic compounds, total anthocyanins, total carotenoids, and antioxidant activity than common chickpeas. Black and brown chickpeas have more polyunsaturated fatty acids, including essential fatty acids [10].

The extraction of aquafaba from beans can follow a simple procedure. Aquafaba is the extract obtained from the usual cooking of beans without pre-processing. The hypothesis is that the extraction efficiency of this valuable substance can increase if pretreatments, for example, mechanical assist-processes have used in aquafaba extraction process [[11], [12], [13]]. Ultrasound waves application have fundamental advantages over other analytical methods and techniques for controlling food processing because many conventional methods are destructive and time-consuming [14,15]. They require much labor, many samples, and are less efficient. Ultrasound waves are accurate, safe, and operate quickly and non-destructively during food processing [16]. It facilitates the analysis of food characteristics and quality, even in concentrated foods, increases extraction efficiency, and may reduce some production costs [17,18]. The ultrasound assisted extraction method is simple, fast, inexpensive, and more efficient than the usual extraction methods. It has wide applications in extracting bioactive compounds from different sources and has high reproducibility while being suitable for a wide range of particle sizes. It significantly reduces the time required for extraction, with a low level of energy consumption, and highly improves the efficiency of polar solvents [19,20]. The only concern about extraction with waves is the destructive effect of waves on the chemical structure of the desired substance [21].

The explosion and disintegration of the bubbles resulting from ultrasound cause physical, chemical, and mechanical changes, so the plant tissue partly disintegrates. The solvent penetration into the tissue improves and increases the release of target compounds inside the solvent. Many researchers have used this method to extract plant polysaccharides or proteins. Along with high-speed extraction, the material extracted with ultrasound showed good yield, purity, emulsion stability, consistency coefficient, color, and molecular weight, which proves that this process has good potential for industrial use [21].

Previous studies showed that researchers investigated the effect of ultrasound on the physicochemical characteristics of aquafaba from various legumes [12,13]. None of the previous studies have investigated the effects of this treatment on extraction efficiency. Aquafaba has not received much research attention to realize its high-added value. Despite conducting various research on the physicochemical properties of aquafaba from beans, no volume of research has addressed the properties of black chickpeas aquafaba as one of the cultivars with high nutritional value. Therefore, this research aimed to optimize the extraction of aquafaba from black chickpeas with assistant of ultrasound waves as pre-treatment and to investigate its physicochemical properties.

## 2 Materials and methods

Black chickpeas were purchased from Abgineh company in Isfahan city, Iran. The moisture content of the black chickpeas was 10.2 % (w.b) and was stored in a cold storage at a temperature of 4 °C until use.

### 2.1 Ultrasonication of black chickpea seeds as pre-processing

First, chickpea seeds were mixed with water at a ratio of 1:4 and soaked for 16 h. Then, the black chickpeas seeds were washed three times with tap water. The soaked chickpea seeds were added into distilled water at a ratio of 1:3. A 10 mm titanium ultrasound probe (UP200Ht Hielscher, Germany) was placed in the mixture for 10, 20 and 30 min, and at amplitude of 50, 70 and 90 % (at response surface method (RSM)) [13]. The samples were sonicated continuously in an ice bath to prevent temperature increase [22].

### 2.2 Aquafaba extraction from black chickpeas

After applying the ultrasound pretreatment according to the mentioned conditions, the black chickpeas seeds were cooked in a pressure cooker for 30 min and the cooked black chickpeas seed were used for the ultrasonication pre-process. Then, the mixture of ultrasonicated black chickpeas seeds and their cooking water were transferred to a glass container and kept them in a refrigerator (5 °C) for 24 h. After 24 h, the cooking water was separated from the cooked chickpeas. the separated cooking water was regarded as aquafaba liquid for further experiments [13].

### 2.3 Techno-functional characteristics of ultrasound pre-processed aquafaba

#### 2.3.1 Aquafaba extraction efficiency

The aquafaba extraction efficiency was calculated according to Alsalman et al. [23] by dividing the aquafaba amount obtained from cooking black chickpeas by the chickpea amount consumed. According to the following equation, the results appeared as grams of aquafaba per 100 g of black chickpeas:

(1) $$Yield\;\left(\%\right)=\frac{Q_{a}}{Q_{c}}\times 100$$

where Qa was the aquafaba amount (g), Qc was the black chickpeas consumed (g).

#### 2.3.2 Measurement of soluble protein content

The amount of soluble protein was determined using the Bio-Rad Protein Assay Kit and the Bradford method based on the spectrophotometric technique [23]. In this method, standard curve was first prepared from the absorbance value at 595 nm plotted against bovine serum albumin protein (as a standard) concentration (0–2 mg/mL). Aquafaba samples were diluted with distilled water to obtain absorbance equivalent of 0.125–2 mg protein. For testing, 40 μl of sample solution was mixed with 2 mL Bradford reagent in cuvette tube, and the absorbance was measured at 595 nm after incubating for 5 min at room temperature.

#### 2.3.3 Black chickpeas aquafaba density

Aquafaba densities were measured using a 25 mL glass pycnometer with pouring slowly to prevent bubble formation. Samples were weighed in an analytical balance (AUW220, Shimadzu, Japan) [13]. Density was calculated using the following equation:

(2) $$\rho =\frac{m}{v}$$

where m was the mass (g) of 25 mL of aquafaba.

#### 2.3.4 Measurement of dry matter content

The percentage of aquafaba dry matter was calculated via the gravimetric method through a hot air circulation oven (Memmert UF110, Germany) [24]. Accordingly, 10 g of aquafaba was poured into a glass plate and dried it in an oven at 105 °C until it reached a constant weight. The dry matter weight relative to 100 g of aquafaba appeared as a percentage of dry matter.

#### 2.3.5 Foaming ability (FA) and foam stability (FS)

The FA and FS properties were determined according to Shim et al. [3] with minor modifications. Exactly 30 mL of aquafaba (V_0_) was poured into a 250 mL beaker and immediately stirred with an IKA overhead stirrer model RW 20. The solution was mixed at 1500 RPM for 2 min until it became foamy. Then, the resultant foam was poured into a 250-graduated cylinder and the initial foam volume (V_1_) was read. After 30 min, the foam volume (V_2_) was reread. The FA and FS were calculated by the following equations:

(3) $$FA=\frac{V_{1}}{V_{0}}\times 100$$

(4) $$FS=\frac{V_{2}}{V_{1}}\times 100$$

### 2.4 Experimental design and optimization of ultrasound pre-processed aquafaba

An experimental design as a rotatable central composite (RCCD) by the response surface method (RSM) determined the effect of ultrasonication time and amplitude on the response variables of extraction efficiency, soluble protein content, density, dry matter, FA, and FS. four replicates at the center point were used for estimation of a pure error sum of squares. Independent variables included ultrasonication time (10–30 min) and amplitude (50–90 percent). Thirteen experimental operations ran randomly and determined the experiments in three replications. The experimental data can fit into the model via a response surface polynomial model. The following mathematical model enabled the prediction of results:

(5) $$Y=\beta_{0}+\sum_{i=1}^{3}\beta_{i}X_{i}+\sum_{i=1}^{3}\beta_{ii}X_{i}^{2}+\sum_{i=1}^{2}\sum_{j=i+1}^{3}\beta_{ij}X_{i}X_{j}$$

where *Y* is the predicted response, β_0_ is the offset term of the system, β_i_, β_ii_, and β_ij_ are the linear, quadratic, and reciprocal regression coefficients, and X_i_, X_i_^2^, and X_i_X_j_ are linear, quadratic, and interaction effects of the independent variables, respectively [25]. The results were analyzed through a multiple regression method with a backward elimination pattern. Thus, it removed insignificant values (p < 0.05) from the regression model. Design-Expert software (version 12.0.3) (Stat-Ease Inc., Minneapolis, U.S.A) analyzed the results and determined the best model for fitting the data on the response surface charts. The upper, lower, and middle limits per independent variable were described and the optimum extraction conditions were achieved.

### 2.5 Fourier transform infrared spectroscopy (FT-IR)

The FT-IR analysis was used for evaluating and comparing the black chickpeas aquafaba ingredients and structure under ultrasonication pre-processing. First, the aquafaba samples were frozen at −20 °C and then lyophilized using a freeze dryer (Alpha 2–4 LD Plus, Germany) at −30 °C and 13 Pa vacuum. The black chickpeas aquafaba lyophilized powders were ground with potassium bromide (KBr) and then pressed into pellets before measurement. The FT-IR operated using a Bruker Tensor II FT-IR device at the frequency range of 400–4000 cm^−1^ at a resolution of 1.43 cm^−1^ [26].

### 2.6 Thermogravimetric analysis (TGA)

Thermal analysis was performed with a TGA-DSC thermogravimetric device (Mettler Toledo, Switzerland) under nitrogen (N_2_) pressure. Approximately 5–7 mg of lyophilized black chickpeas aquafaba powder was placed in the device and analyzed in the temperature range of 25–600 °C with a temperature rate of 10 °C per minute.

## 3 Results and discussion

### 3.1 Model fitting and data analysis

The calculation of extraction efficiency was possible by measuring aquafaba protein, density, dry matter, FA, and FS during the extraction process. Ultrasonication time and amplitude affected the mentioned variables. The most suitable model appeared in calculations to fit the data with the backward elimination. In this method, the several factors were added to the model, i.e., response mean, linear effects, interaction effects, and quadratic effects. Then, their significance in the model was tested. The results showed that the quadratic model optimally evaluated how each factor affected changes in the variation trend of response variables. The significance of all main effects, linear, interaction, and quadratic effects for each dependent variable are in Table 1.

**Table 1:** ANOVA and regression coefficients of the second-order polynomial model for the response variables.

source	extraction efficiency			protein content			Density		
	Coefficient	SS	p-Value	Coefficient	SS	p-Value	Coefficient	SS	p-Value
Model	120.70042	719.97	0.0097	7405.92430	6.121 × $10^{6}$	0.0010	0.936629	0.0007	0.0128
Linear									
A	0.482449	186.21	0.0171	6.26629	1.346 × $10^{6}$	0.0038	0.000771	0.0004	0.0031
B	0.370433	439.11	0.0014	265.82824	1.082 × $10^{6}$	0.0066	–	–	ns
Quadratic									
AA	–	–	ns	–	–	ns	–	–	ns
BB	–	–	ns	1.75156	3.415 × $10^{6}$	0.0003	−0.000016	0.0003	0.0070
Interaction									
AB	–	–	ns	–	–	ns	–	–	ns
Lack of fit			0.01705 ^ns^			0.0959 ^ns^			0.2863 ^ns^
$R^{2}$	0.8435			0.9216			0.8296		
Adj-$R^{2}$	0.7317			0.8655			0.7079		
CV	2.11			10.30			0.4367		

**source**	**dry matter**			**FA**			**FS**		
	Coefficient	SS	p-Value	Coefficient	SS	p-Value	Coefficient	SS	p-Value
Model	3.06774	1.06	0.0019	−6.54183	5164.10	0.0205	63.33435	231.01	0.037
Linear									
A	0.023183	0.4300	0.0097	2.75260	2720.80	0.0061	–	–	ns
B	0.013988	0.6262	0.0032	–	–	ns	–	–	ns
Quadratic									
AA	–	–	ns	–	–	ns	0.056189	219.63	0.0024
BB	–	–	ns	–	–	ns	–	–	ns
Interaction									
AB	–	–	ns	–	–	ns	–	–	ns
Lack of fit			0.1527 ^ns^			0.1703 ^ns^			0.4680 ^ns^
$R^{2}$	0.8752			0.8030			0.7628		
Adj-$R^{2}$	0.7861			0.6623			0.5934		
CV	3.60			6.23			7.48		

ns: no significant.

CV: coefficient of variation; A: Sonication time; B: Amplitude.

The coefficient of determination (R^2^) and the lack of fit test enabled the process to check the correctness of the model. The coefficient of determination value (R^2^) changed between zero and one. The closer this number is to one, it means that the model fits almost all the data with high accuracy. When the R^2^ is above 0.8, the model may be a fit for the data. R^2^ describes extraction efficiency, aquafaba protein, density, dry matter, FA, and FS, which were 0.8435, 0.9216, 0.8296, 0.8752, 0.8030, and 0.7628, respectively. This high R^2^ value shows that the changes were calculated in the model, and the data were well fitted with the quadratic polynomial model. The lack of fit test for all measured traits was not significant (p ≤ 0.05), which means the model fitted the investigated data well. The coefficient of variation (CV) and the adjusted coefficient of determination (Adj-R^2^) (Table 1) ensured that the presented models have the adequacy to fit and estimate the data.

The Adj-R^2^ for extraction efficiency, aquafaba protein, density, dry matter, FA, and FS were 0.7317, 0.8655, 0.7079, 0.7861, 0.6623, and 0.5934, respectively. Adj-R^2^ values should be close to R^2^ values to confirm the correctness of the model. The results showed that R^2^ and Adj-R^2^ values were not significantly different regarding all the attributes on which the effect of the variables was significant, except for density, FA, and FS. Since adding variables to the model always increases R^2^, the closeness of R^2^ and Adj-R^2^ means that non-significant variables have not added into the model irrationally. The coefficient of variation (CV) shows the amount of numerical dispersion around the points on the model for extraction efficiency, aquafaba protein, density, dry matter, FA, and FS were 1.71, 6.3, 0.6743, 3.60, 5.43, and 7.48, respectively. The lower the CV, the higher the reproducibility.

### 3.2 Black chickpeas aquafaba extraction efficiency

**Fig. 1:**
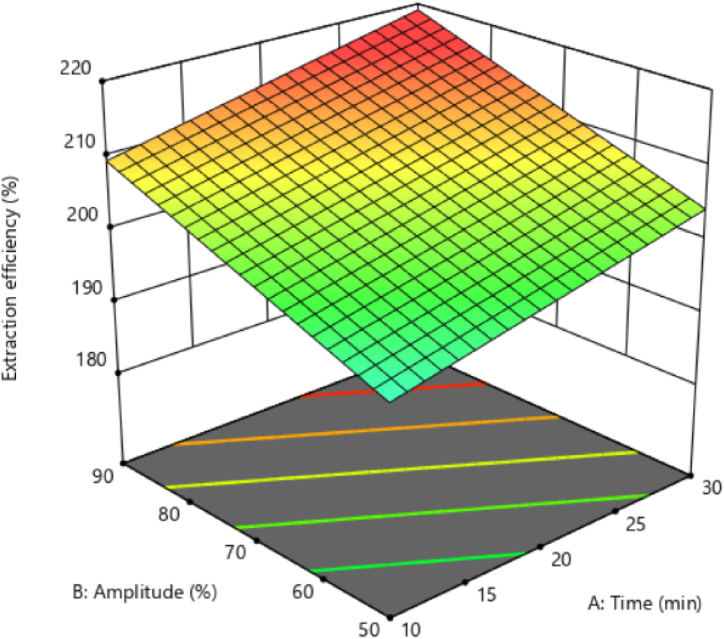
Extraction efficiency of black chickpeas aquafaba under ultrasonication process.

The results showed that the linear effect of ultrasound time and amplitude significantly affected the extraction efficiency (p < 0.05). According to the sum of squares, the amplitude was the most important in extraction efficiency (Table 1). Changes in aquafaba extraction efficiency concerning ultrasound time and ultrasound amplitude (Fig. 1) showed that by increasing the ultrasound time, the efficiency of aquafaba extraction increased significantly (p < 0.05). Also, the efficiency of aquafaba extraction increased significantly through an increase in the ultrasound amplitude. Romdhane and Gourdon [27] reported that ultrasound improved the kinetics and extraction efficiency of pyrethrines from *Chrysanthemum cineraria*. They attributed the increase in extraction efficiency to a higher internal diffusion rate, which increased the migration of components into the solvent. Also, they reported that ultrasound waves create pores in tissues through cavitation and destruction of plant structure, thereby releasing trapped compounds [27].

### 3.3 Black chickpea's aquafaba protein

**Fig. 2:**
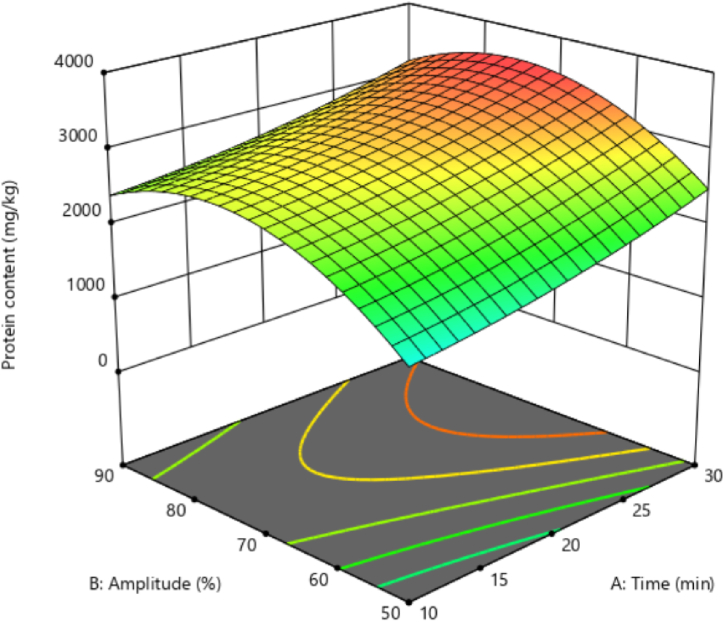
Protein content of black chickpeas aquafaba under ultrasound assisted extraction.

Soaking and cooking reduces the protein content in dry beans, and its reduction can be related to the discharge of protein into the cooking water. The FA of the extract, resulting from soaking or cooking beans, mainly results from the protein and albumin amount in them [28]. The results showed that the quadratic effect of the ultrasound amplitude was significant on the amount of aquafaba protein extracted from black chickpeas. Linear and interaction effects in none of the variables were significant. Fig. 2 shows changes in protein content in response to the ultrasound time and amplitude. The results showed that increasing the ultrasound time increased the protein content in aquafaba extracted from black chickpeas. Also, an increase in ultrasound amplitude from 50 % to 70 % enhanced the aquafaba protein content. But further increasing the ultrasound amplitude from 70 % to 90 % caused a slight decrease in protein content (Fig. 2). Using high amplitude levels can lead to partial degradation and denaturation of soluble proteins. This, in turn, may reduce the amount of soluble proteins extracted from plant tissues [22].

Kilicli and Toker [12] subjected the chickpea seeds to ultrasound treatment at the soaking stage. They continued cooking and extracting aquafaba after removing the soaked water and adding fresh water. They reported that the protein content of the aquafaba sample decreased with the application of ultrasound in the soaking stage of processing of chickpeas. This decrease in protein was probably due to structure damage caused by ultrasound and increased protein solubility, resulting in their release via ultrasound while soaking in water. In current study, the same soaked water served as the solvent for ultrasound treatment and aquafaba production. Thus, the protein content increased in the extracted aquafaba in response to longer ultrasound durations. However, the protein content probably denatured in response to high ultrasound amplitude and decreased solubility.

### 3.4 Black chickpea's aquafaba density

The effect of ultrasonication time on aquafaba density was significant (p < 0.05). The ultrasound amplitude had no substantial impact on aquafaba density. No significant interaction and third-order effects among the dependent variables affected the density. Among the quadratic effects of independent variables, the ultrasound amplitude substantially affected density (p < 0.05).

**Fig. 3:**
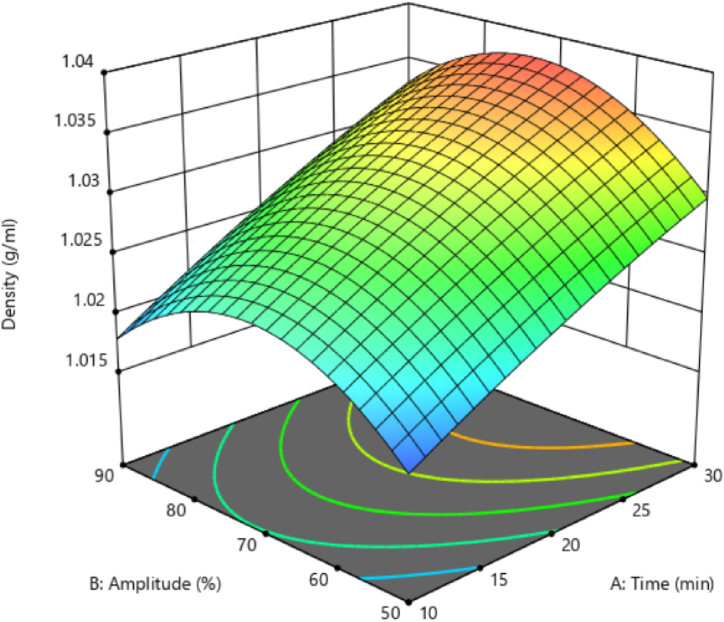
Black chickpeas aquafaba density in different ultrasonication time and amplitude.

Fig. 3 shows changes in density regarding ultrasound time and amplitude. The results showed that increasing ultrasonication time enhanced the density from 1.01576 to 1.0382. An increase in ultrasound amplitude initially enhanced the density but then decreased it. The aquafaba density in different legumes is almost similar. Almost equal amounts of insoluble carbohydrates (0.93–2.46 g/100 g) can be responsible for this density. These components are mainly polysaccharides (not starch), cellulose, hemicellulose, lignin, or pectin, related to the structure of the legume cell wall [29].

In this regard, Hematian Sourki et al. [21] reported that ultrasound waves create cavitations by generating gas bubbles inside the liquid and then exploding these bubbles. The explosion and rupture of these bubbles produce a lot of energy that causes extensive physical, chemical, and mechanical changes in the texture of the treated food. Due to cavitation and energy release phenomena, plant tissues experience extensive damage. Thus, the solvent penetration rate into the tissue improves and increases the release of target compounds in the solvent. Accordingly, solid particles in the solution likely diffuse into the solvent when prolonging the ultrasound duration, thereby increasing the aquafaba density.

### 3.5 Dry matter content of black chickpea aquafaba

The dry matter of legumes’ cooking water (aquafaba) mainly contains protein, soluble carbohydrates, insoluble carbohydrates, ash, saponin, and phenolic compounds [30,31]. The analysis of variance showed that the linear effect of both ultrasonication time and amplitude substantially determined the amount of aquafaba dry matter from black chickpeas. The ultrasound amplitude was most effective on the dry matter content, referring to the sum of squares (Table 1). Also, the results showed that the interaction and second-order effects of independent variables did not significantly affect the amount of dry matter.

**Fig. 4:**
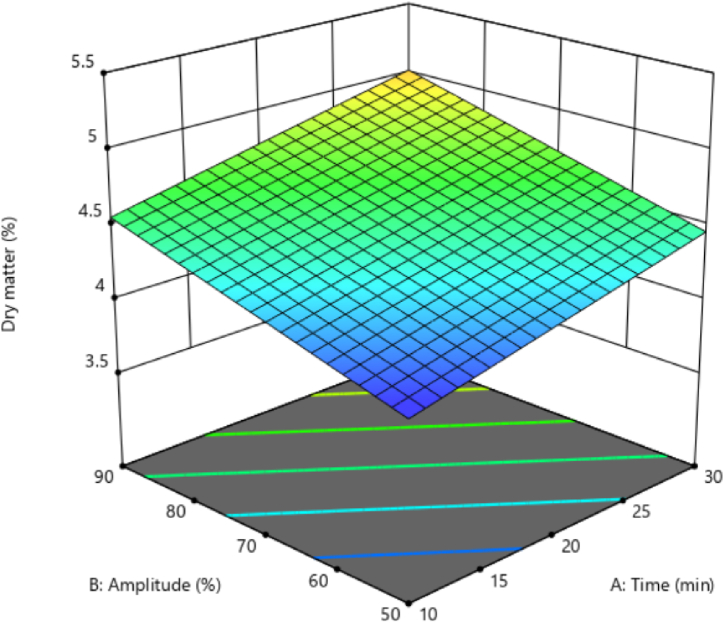
Dry matter of black chickpeas aquafaba extracted by ultrasonication process.

Fig. 4 shows the changes in the amount of dry matter concerning ultrasonication time and amplitude. The results showed that increasing ultrasonication time enhanced the dry matter content. Also, as seen in Fig. 4, an increase in the ultrasound amplitude caused the dry matter content to increase significantly (p < 0.05). Increasing the ultrasonication time and amplitude caused a destructive effect of the cavitation phenomenon on the texture of black chickpeas. Thus, the penetration of the solvent and the release of solvent solid compounds leveled up regarding components such as carbohydrates, proteins, and minerals from inside the texture of black chickpeas. Their transfer to the solvent increased the soluble solid compounds and the aquafaba dry matter content. Kutlu et al. [32] reported that an increase in heat and mass transfer occurred with the collapse of cell walls due to cavitation, which is highly important in increasing the density and dry matter content of aquafaba extracted with ultrasound.

### 3.6 The FA of black chickpeas aquafaba

Foams are colloidal systems consisting of air bubbles dispersed in a liquid, in this case, aquafaba. This liquid separates the adjacent gas bubbles into thin soluble layers called lamellae. Foam formation occurs by stirring the solution via a mechanical action. Percussive stirring causes the air to remain in the form of large bubbles in the viscous liquid. With a long stirring time, the bubbles turn smaller and enhance foam volume. The diameter of foam bubbles usually varies from 1 μm to several centimeters. Foaming agents are aquafaba solid particles (especially proteins, polysaccharides, and saponins). There is a strong correlation between aquafaba protein content and FA. As we know, proteins are the main foaming agents that stabilize foams. Therefore, the higher the protein concentration in aquafaba, the higher the foaming properties [29].

**Fig. 5:**
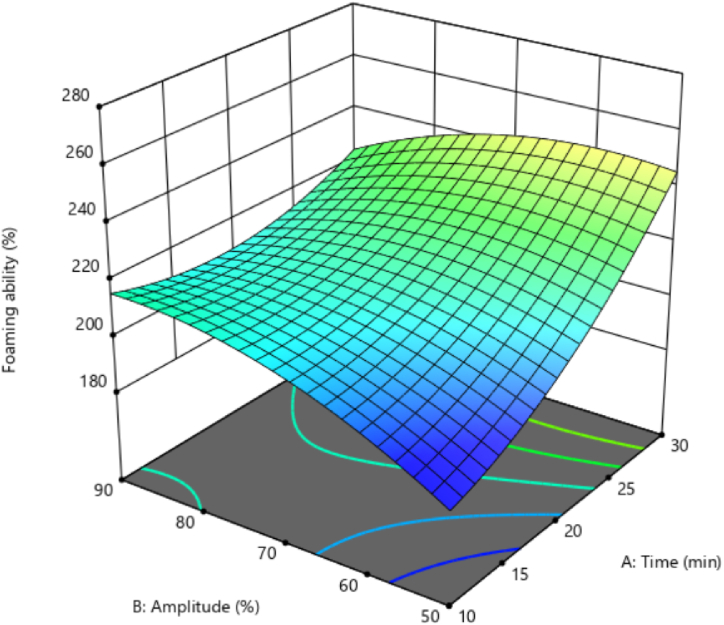
The effect of ultrasonication pre-process on foaming ability of black chickpeas aquafaba.

The analysis of variance showed that, among the linear effects of independent variables, ultrasonication time significantly affected the FA. However, the linear effects of ultrasound amplitude, interaction and second-order effects from none of the independent variables affected FA significantly (p < 0.05). The FA per the independent variables of ultrasound time and amplitude (Fig. 5) showed that the FA increased significantly in low ultrasound amplitude by increasing the ultrasonication time. However, the parallel increase in ultrasonication time and FA was not observed when using high ultrasound amplitudes. Increasing the ultrasonication time while having high amplitudes resulted in an adverse effect on FA. In other words, a lengthier ultrasonication time in combination with high ultrasound amplitudes decreased the FA. The decrease in FA at high amplitude levels may be due to the destructive effect of the energy emitted from the ultrasound processor on the protein. Thus, the pressure and high energy caused by cavitation prompted parts of the hydrophobic bonds in the protein structure to break, thereby reducing the ability to connect proteins with air molecules [12].

According to Fig. 5, at low ultrasound durations (10 min), an increase in ultrasound amplitude enhanced the FA. Further increasing the ultrasonication time had the opposite result. As can be seen, the highest FA occurred in low ultrasound amplitude and lengthy ultrasound time or high ultrasound amplitude and low ultrasound time (Fig. 5).

Meurer et al. [13] reported that an ultrasound treatment with an appropriate ultrasound time and amplitude may enhance aquafaba quality extracted from peas, thereby increasing the foaming capacity and foam stability. They believe that the relative denaturation of proteins and the surface exposure of the hydrophobic parts of proteins increases the absorption of proteins at the air-water interface and decreases the surface tension. Also, they reported that applying ultrasound treatment on chickpea aquafaba reduced the size of protein-protein aggregates so that protein particles take form more abundantly. Due to their low molecular weight, they are absorbed faster at the interface and increase the FA [13].

### 3.7 The FS of black chickpeas aquafaba

Due to the large surface area of the aqueous phase, the foam has little stability and tends to break due to liquid settling. Gravitational forces cause the liquid to drain and decrease FS. In addition, since air is essentially non-polar, there is an order in aquafaba molecules that increases surface tension and system energy. These two facts mean that the foam is also thermodynamically unstable. Its decomposition reduces the free energy within the system. Therefore, the stabilizing agent is necessary here to increase the FS [29].

**Fig. 6:**
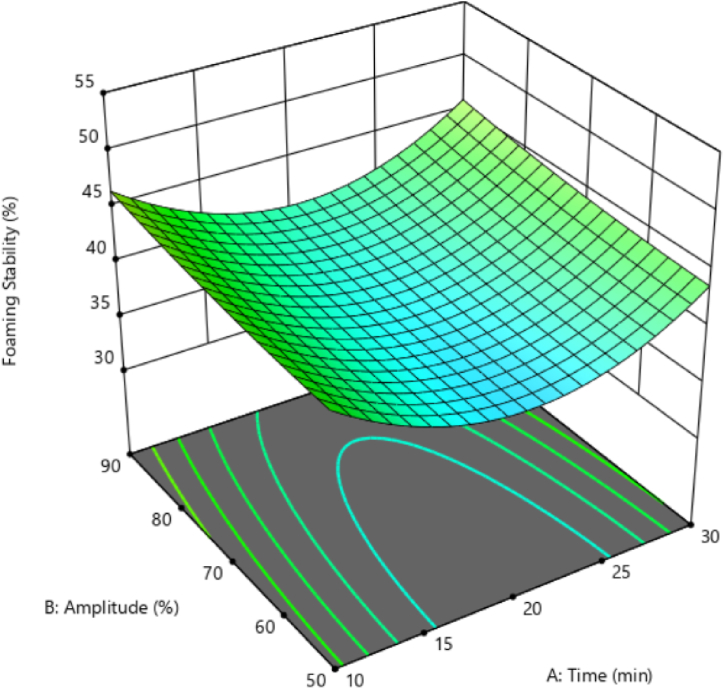
Foaming stability of black chickpeas aquafaba under ultrasonication pre-process.

The analysis of variance showed that the linear, interaction, and third-order effects of ultrasound time and amplitude affected FS insignificantly. Among the quadratic effects, the variable effects of ultrasound time on FS were significant. Fig. 6 shows the changes in FS in response to ultrasound time and amplitude. The results showed that FS increased, parallel to higher levels of ultrasound amplitude. Also, according to Fig. 6, the FS decreased by increasing the time of ultrasound from 10 to 20 min. The FS increased in response to lengthier durations of ultrasound application from 20 to 30 min.

Foam with large bubbles tends to deflate or form a cream. The main reason for the breaking of foam is the decrease in the thickness of the lamellae (critical point: thickness 5–15 nm), which causes coalescence. This reduction in thickness may be due to gravity, deformation forces exerted by gas movements (diffusion), or capillary drainage. Upon reaching maximum foam capacity, over-stirring becomes counterproductive. The FS declines due to excessive protein expansion that leads to the coagulation of proteins at the air-water interface. This coagulation leads to the loss of water-holding capacity [29].

Cavitation during ultrasound treatment breaks hydrogen and hydrophobic bonds, decreasing the protein molecular weight and increasing the interaction between protein and water molecules [33,34]. Ampofo and Ngadi [35] stated that the longer duration and amplitude of ultrasound treatment leads to the formation of protein aggregates, or more hydrophobic groups become exposed to the surface. In effect, it reduces protein solubility. On the other hand, protein solubility indicates the degree of protein denaturation and aggregation, which affects other main techno-functional properties such as foam formation and emulsion [[36], [37], [38]]. Therefore, lengthier ultrasound durations prompt the denaturing of more proteins, more hydrophobic surfaces come into contact with the surface, and the FS increases.

### 3.8 Optimization of aquafaba extraction from black chickpeas

Numerical optimization conditions of aquafaba extraction resulted from calculations of maximum extraction efficiency, protein content, FA, and FS. All variables were similar to optimize extraction conditions of aquafaba with ultrasound. The importance of the variables developed according to the purpose of the research, that is, to extract aquafaba with the highest extraction efficiency, FA, and FS. The factors aimed to maximize values of the response variables, i.e., extraction efficiency, protein content, FA, and FS. Density and dry matter appeared within the expected range for the response variables. The results showed that the optimal conditions for extracting aquafaba from black chickpeas included an ultrasound time of 30 min and an ultrasound amplitude of 72 % (Table 2). According to these conditions, the highest extraction efficiency was 212.07 %, the protein content was 3546.7 mg/kg, the density was 1.038 g/mL, the dry matter was 4.76 %, the FA was 243.57 %, and FS was 44.50 %.

**Table 2:** Predicted optimum condition for ultrasonication pre-process of black chickpeas aquafaba.

Independent variables	low	high	optimum
Ultrasonication time (min)	10	30	30
Amplitude (%)	50	90	72

### 3.9 Fourier transform infrared spectroscopy

**Fig. 7:**
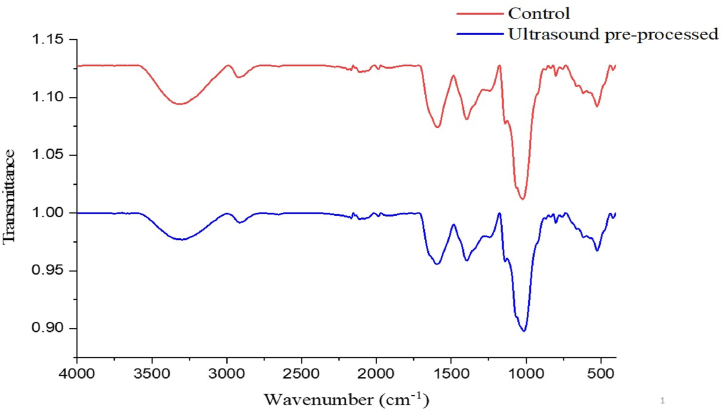
FT-IR analysis of pre-processed black chickpeas under optimum condition of ultrasonication.

The FT-IR spectrum of aquafaba preprocessed with optimal conditions of ultrasonication and control sample (aquafaba extracted without ultrasonic pre-treatment) are illustrated in Fig. 7. The results showed that the absorption spectrum of aquafaba pretreated with ultrasound had sharp peaks in wave numbers 1014, 1242, 1395, 1595, 2913, 3298 cm^−1^. Also corresponding to these wave numbers, the control aquafaba had peaks in wave numbers 1022, 1139, 1395, 1591, 2916, 3301 cm^−1^. The broad peak that appeared in the range of 3000–3500 cm^−1^ was related to the O─H stretching vibration caused by the presence of starch and protein-starch interactions, as well as the presence of various compounds such as alcohol, phenols and carboxylic acids [39]. The absorption peaks at wave numbers of 2913 (treated sample) and 2916 cm^−1^ (control sample) indicated the presence of amines that usually exist in the internal matrix of black chickpeas in different forms such as peptides, amino acids, proteins, DNA, RNA and alkaloids. The absorption peaks in the area of 1400–1600 cm^−1^ are related to N-H bending vibrations of type I amines. Also, the presence of absorption peaks in the region of 800–1200 cm^−1^ is related to bending C-H out-of-plane on an aromatic ring [40]. Absorption peaks in the ranges of 2800–3050, 1550–1750, 1400–1660 and 900-1200 cm^−1^, corresponding to vibrations caused by symmetric and asymmetric stretching CH of triglycerides [41], amides I and II of proteins, bending vibrations of N-H and stretching C=O [12] and the presence of carbohydrates [41], respectively.

According to Fig. 7, no major difference was observed between the FT-IR spectrum of the control sample and the pretreated sample with optimal ultrasound conditions. In fact, almost all the peaks in the FT-IR spectrum of the control sample were observed in the spectrum of the pre-treated sample with optimal ultrasound conditions with a slight shift, which indicates the absence of the destructive effect of ultrasound pre-process on the structure of the chemical compounds of aquafaba and the absence of the formation of unknown or harmful compounds [42].

### 3.10 Thermal properties

**Fig. 8:**
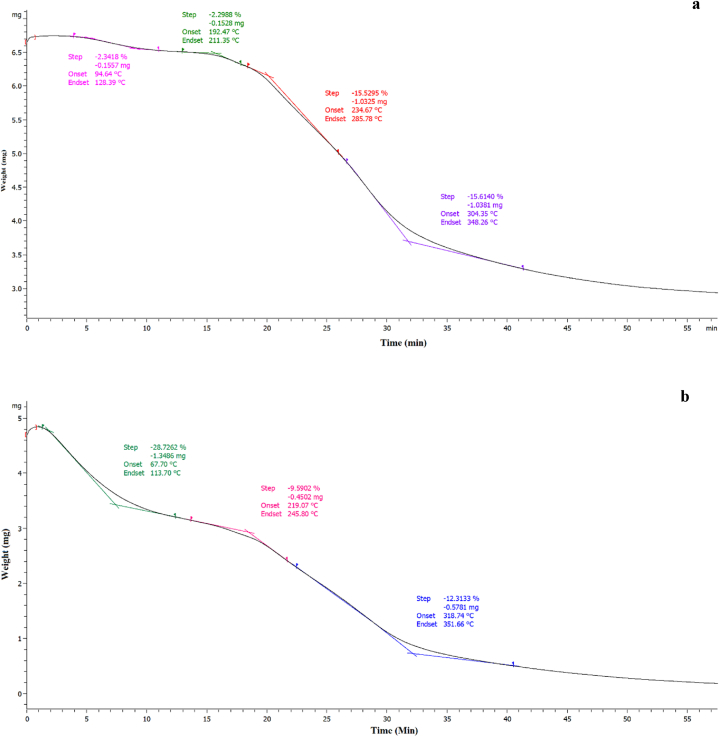
TGA analysis of pre-processed black chickpeas under optimum condition of ultrasonication (a) compared to control sample (b).

TGA curves can directly show the details of the mass loss of samples during heat treatment, which provides valuable information about the material's thermal stability and molecular interactions [43]. According to the TGA curves of the sample pre-processed with ultrasound and the control sample, it can be seen that the first zone of weight loss in both samples is related to the loss of adsorbed and bound water (Fig. 8). The first zone in the TGA curve of the control sample is more intense and occurred at a lower temperature, which can be due to less protein accumulation in this sample and as a result, its higher water binding capacity. In other studies, generally, the first zone of weight loss of the samples is related to the evaporation of water [[44], [45], [46], [47], [48]].

The second zone observed in the sample pre-treated with ultrasound (192–211 °C) may be related to lipid decomposition, which is due to the significant difference between the lipid content of the treated sample and the control (results not shown) this peak was not observed in the control sample curve. Raza et al. (2019) attributed the second zone, between 110 and 250 °C, mainly to the degradation of peanut protein powder fats. Huntrakul et al. [49] have attributed the occurrence of weight loss in the temperature range of 197.75–193.75 °C to fatty acid evaporation.

The third zone observed in the TGA curve of the sample pretreated with ultrasound (234–285 °C) and the second zone in the TGA curve of the control sample (219–245 °C) are probably related to the breaking of S-S, O-N and O-O bonds in protein molecules and the initiation of the breakdown of proteins [50]. The higher temperature of decomposition and destruction of proteins in the pre-processed sample with ultrasound is probably due to the accumulation of proteins due to ultrasound waves and as a result, increasing their thermal stability. Malik et al. [44] and Li et al. [45] have also attributed the weight loss in this temperature range to the breakdown of proteins and the breaking of non-covalent or covalent bonds in the structure of proteins. The last zone in the TGA curve of the pre-treated sample with optimal ultrasound conditions (304–348 °C) and the TGA curve of the control sample (318–351 °C) are related to the complete decomposition of proteins [50].

In general, it can be concluded that the pre-sonication of aquafaba can increase the thermal stability of this compound and therefore facilitate the use of this substance in foods that are processed at high temperatures.

## 4 Conclusion

In general, this research showed that ultrasound assisted extraction (UAE) significantly affected the extraction efficiency, the amount of soluble protein, and the physicochemical properties of aquafaba obtained from black chickpeas (p < 0.05). The results showed that with the increase in the sonication time and amplitude, the extraction efficiency, soluble protein content, the density, and dry matter content of black chickpeas aquafaba increased significantly (p < 0.05). The results also demonstrated that FA (in short ultrasonication times) and FS significantly increased with higher amplitude. The results of numerical optimization of ultrasound application showed that ultrasound application for 30 min at 72 % amplitude on black chickpeas before cooking created the best conditions for aquafaba extraction. The highest extraction efficiency (212.07 %), protein content (3546.7 mg/kg), density (1.038 g/mL), dry matter (4.76 %), FA (243.57 %), and FS (44.50 %) occurred in these conditions. In general, this research showed that black chickpeas are a very valuable source for the production of aquafaba, and with the help of ultrasonic waves, we can anticipate the production of large aquafaba quantities from the waste and wastewater of cooking black chickpeas in the canning industry. Thus, aquafaba can be used as a high-added value material in diverse food industries.

## CRediT authorship contribution statement

**Fatemeh Roosta:** Writing – original draft, Methodology, Formal analysis, Data curation. **Abdollah Hematian Sourki:** Writing – review & editing, Writing – original draft, Validation, Supervision, Resources, Project administration, Methodology, Investigation, Conceptualization.

## Ethical statement

This research did not involve the use of human and animal subjects.

## Consent for publication

All authors listed have read the complete manuscript and have approved submission of the paper.

## Availability of data and material

The datasets used and/or analyzed during the current study are available from the corresponding author on reasonable request.

## Funding

Not applicable.

## Declaration of competing interest

No conflicts of interest declared by the authors.

## References

[1] Nguyen T.M.N., Nguyen T.P., Tran G.B., Le P.T.Q. (2020). J. Food Process. Preserv..

[2] Zain Despal M., Tanuwiria U.H., Pazla R., Putri E.M., Amanah U. (2023). Int. J. Vet. Sci..

[3] Shim Y.Y., Mustafa R., Shen J., Ratanapariyanuch K., Reaney M.J.T. (2018). JoVE.

[4] Aslan M., Ertaş N. (2020). Harran tarım ve gıda bilimleri dergisi.

[5] Beeber M., Panitz A., Traynor C., Zanville K., Ghatak R., Bhaduri S., Navder K. (2019). J. Acad. Nutr. Diet..

[6] Buhl T.F., Christensen C.H., Hammershøj M. (2019). Food Hydrocolloids.

[7] Azizi E., Mirbolook A., Behdad A. (2017). J. Crop Prod..

[8] Pavan S., Lotti C., Marcotrigiano A.R., Mazzeo R., Bardaro N., Bracuto V., Ricciardi F., Taranto F., D'Agostino N., Schiavulli A. (2017). Plant Genome.

[9] Costantini M., Summo C., Faccia M., Caponio F., Pasqualone A. (2021). Molecules.

[10] Summo C., De Angelis D., Ricciardi L., Caponio F., Lotti C., Pavan S., Pasqualone A. (2019). J. Food Compos. Anal..

[11] Kilicli M., Özmen D., Bayram M., Toker O.S. (2023). Int. J. Gastron. Food Sci..

[12] Kilicli M., Toker O.S. (2022). Int. J. Food Eng..

[13] Meurer M.C., de Souza D., Ferreira Marczak L.D. (2020). J. Food Eng..

[14] Khalil A.A., Khan A.A., Khalid A., Abid Z., Proestos C., Bhat Z.F., Shahbaz M.U., Aadil R.M. (2023). Ultrason. Sonochem..

[15] Roobab U., Abida A., Madni G.M., Ranjha M.M.A.N., Zeng X.-A., Mousavi Khaneghah A., Aadil R.M. (2023). Journal of Agriculture and Food Research.

[16] Mukhtar K., Nabi B.G., Arshad R.N., Roobab U., Yaseen B., Ranjha M.M.A.N., Aadil R.M., Ibrahim S.A. (2022). Ultrason. Sonochem..

[17] Reddy I., Seib P. (2000). J. Cereal. Sci..

[18] HerCeg I.L., Jambrak A.R., ŠubArIć D., Brnčić M., Brnčić S.R., Badanjak M., Tripalo B., Ježek D., Novotni D., Herceg Z. (2010). Czech J. Food Sci..

[19] Chen W., Wang W.-P., Zhang H.-S., Huang Q. (2012). Carbohydrate Polymers.

[20] Zhang Z.-S., Wang L.-J., Li D., Jiao S.-S., Chen X.D., Mao Z.-H. (2008). Separation and Purification Technology.

[21] Hematian Sourki A., Koocheki A., Elahi M. (2017). Int. J. Biol. Macromol..

[22] Wang F., Zhang Y., Xu L., Ma H. (2020). Lwt.

[23] Alsalman F.B., Tulbek M., Nickerson M., Ramaswamy H.S. (2020). Legume Science.

[24] AOAC (1990).

[25] Box G.E., Wilson K.B. (1992). Breakthroughs in Statistics: Methodology and Distribution.

[26] Ali S., Khatri Z., Oh K.W., Kim I.-S., Kim S.H. (2014). Macromol. Res..

[27] Romdhane M., Gourdon C. (2002). Chem. Eng. J..

[28] Serventi L. (2020).

[29] Solé Lamich L. (2022).

[30] He Y., Meda V., Reaney M.J.T., Mustafa R. (2021). Trends Food Sci. Technol..

[31] Mustafa R., Reaney M.J.T. (2020). Food Wastes and By‐products.

[32] Kutlu N., Pandiselvam R., Kamiloglu A., Saka I., Sruthi N., Kothakota A., Socol C.T., Maerescu C.M. (2022). Ultrason. Sonochem..

[33] Resendiz-Vazquez J., Ulloa J., Urías-Silvas J., Bautista-Rosales P., Ramírez-Ramírez J., Rosas-Ulloa P., González-Torres L. (2017). Ultrason. Sonochem..

[34] Zhang Z., Regenstein J.M., Zhou P., Yang Y. (2017). Ultrason. Sonochem..

[35] Ampofo J., Ngadi M. (2022). Ultrason. Sonochem..

[36] Alvarez-Ossorio C., Orive M., Sanmartín E., Alvarez-Sabatel S., Labidi J., Zufia J., Bald C. (2022). ACS Food Science & Technology.

[37] Huang L., Ding X., Li Y., Ma H. (2019). Food Chem..

[38] Liu Y., Ma X.-Y., Liu L.-N., Xie Y.-P., Ke Y.-J., Cai Z.-J., Wu G.-J. (2018). Food Science and Technology.

[39] Kumar Y., Sharanagat V.S., Singh L., Mani S. (2020). Legume Science.

[40] Raza H., Zaaboul F., Shoaib M., Ashraf W., Hussain A., Zhang L., Soci J. Glob Inno Agri (2019). Sci.

[41] Medhe S.V., Kamble M.T., Kettawan A.K., Monboonpitak N., Kettawan A. (2022). Foods.

[42] Hematian Sourki A., Roozitalab R., Ghani A. (2023). J. Food Meas. Char..

[43] Zhang J., Ying D., Wei Y., Zhang B., Su X., Li S. (2017). Journal of Thermal Analysis and Calorimetry.

[44] Malik M.A., Sharma H.K., Saini C.S. (2017). Food Hydrocolloids.

[45] Li N., Qi G., Sun X.S., Wang D., Bean S., Blackwell D. (2014). Transactions of the ASABE.

[46] Chen Q., Zhang J., Liu H., Li T., Wang Q. (2023). Food Hydrocolloids.

[47] Yang K., Xu T.-R., Fu Y.-H., Cai M., Xia Q.-L., Guan R.-F., Zou X.-G., Sun P.-L. (2021). Food Res. Int..

[48] Malik M.A., Saini C.S. (2019). Food Chem..

[49] Huntrakul K., Yoksan R., Sane A., Harnkarnsujarit N. (2020). Food Packag. Shelf Life.

[50] Zhang J., Liu L., Jiang Y., Faisal S., Wei L., Cao C., Yan W., Wang Q. (2019). J. Agric. Food Chem..

